# Management of an Unusual Maxillary Canine: A Rare Entity

**DOI:** 10.1155/2015/780908

**Published:** 2015-12-08

**Authors:** Jaya Nagendra Krishna Muppalla, Krishnamurthy Kavuda, Rajani Punna, Amulya Vanapatla

**Affiliations:** ^1^Kamineni Institute of Dental Sciences, Narketpally, Telangana 508254, India; ^2^Awadh Dental College & Hospital, Jamshedpur, Jharkhand 831012, India

## Abstract

Clinicians need to have intimate knowledge and thorough understanding of both pulp chamber and root canal anatomy. They should be aware of possibility of anatomical variations in the root canal system during endodontic treatment. Maxillary canines usually have single root and root canal but rarely may have single root with two root canals. This case describes a lengthier maxillary canine with two root canals.

## 1. Introduction

Thorough knowledge and understanding of pulp chamber and root canal system anatomy are essential for successful root canal therapy. Familiarity with variations in tooth anatomy and characteristic features in various racial groups can aid location and negotiation of canals [1]. Missed extra root canals are major reason for endodontic failure [2].

Pulp canal system is complex with branching and divisions throughout the root length. Vertucci (1984) classified the root canal configurations of human permanent teeth into various types ranging from single to three separate distinct canals [3].

Permanent maxillary canines are more commonly single rooted, single canal teeth. Presence of two root canals is a rarity [4]. Majority of them join in apical third and exit as single apical foramen [5].

This case report presents a permanent maxillary canine having two root canals exiting as single foramen.

## 2. Case Report

A 35-year-old female patient reported to dental clinic with a chief complaint of spontaneous pain from four days in maxillary left anterior region. Subjective symptoms include sharp, severe, continuous, throbbing pain and are aggravated by taking hot foods and relieved by medication. Past medical history was noncontributory.

Oral examination revealed a deep carious lesion involving maxillary left canine (23, FDI System). The teeth was asymptomatic to palpation and tested negative using electrical pulp tester and gave an exaggerated severe response to cold test. No mobility was seen and tested negative for percussion. Radiographic examination revealed an abnormal root canal morphology. A diagnosis of acute irreversible pulpitis of 23 was established and endodontic treatment was planned.

**Figure 1:**
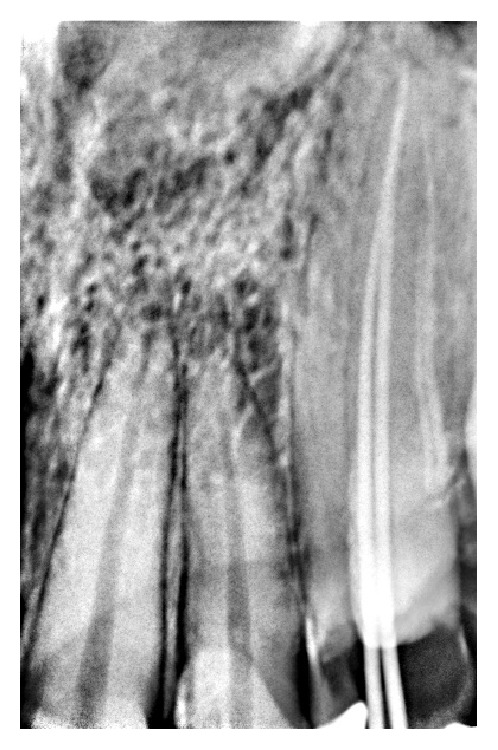
Radiograph showing working length of maxillary canine with two separate canals.

**Figure 2:**
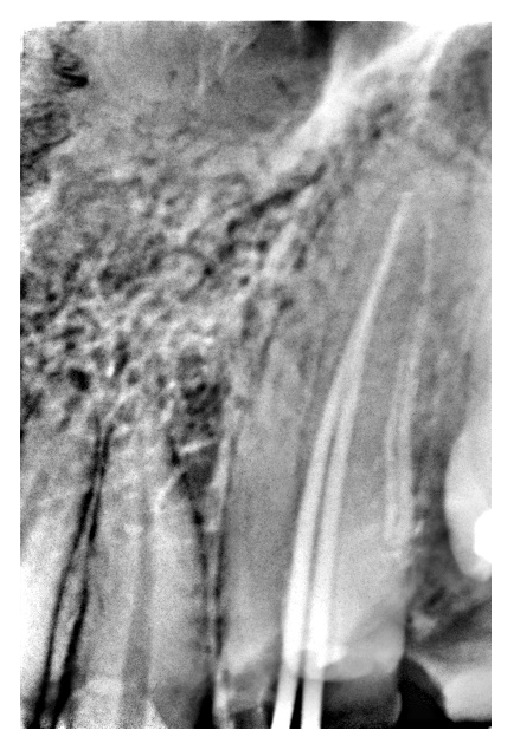
Radiograph showing master cone gutta-percha.

**Figure 3:**
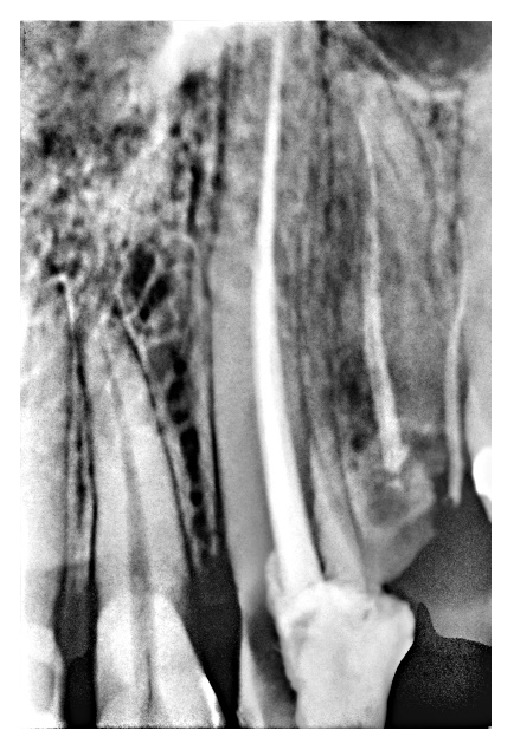
Postoperative radiograph obturated with gutta-percha and AH Plus Sealer.

Following local anesthesia with 2% lidocaine, the tooth was isolated with rubber dam [Hygienic; Coltene Whaledent], and an endodontic access was made on palatal side with #1014 round bur and endo-Z carbide bur. The vital pulp tissue was extirpated and initially two canal orifices were located. Working length of 31 mm was measured by using K flex files #15 of length 31 mm (Figure 1). The palatal canal joined the buccal canal in apical third of root canal (Vertucci type II canal configuration). Crown down root canal preparation was done; the coronal and middle thirds were prepared using Gates Glidden drills #1–3 [Tulsa Dental, Dentsply] and apical preparation by hand K flex files [Dentsply] to size #40. Copious irrigation with 5.25% sodium hypochlorite, 17% EDTA were performed after use of each file. 2% chlorhexidine was used as final irrigant. The canals were dried with paper points and obturated with gutta-percha and AH Plus Sealer (Figure 2) [Dentsply De Trey GmbH, Konstanz, Germany] using a lateral condensation technique (Figure 3). The patient was asymptomatic during the 3-month follow-up.

## 3. Discussion

Debridement of root canal to remove pulpal remnants, bacteria, and their byproducts before obturation is primary requisite for successful endodontic treatment. Being unable to locate and fill a canal results in failure of root canal therapy. Therefore, it is imperative to have knowledge of anatomic variations as endodontic success is related to canal debridement [6].

The diagnostic difficulty and possible canal superimposition on radiographic examination should be kept in mind when examining such cases. When locating extra canals, identification of periodontal ligament space that often projects onto root surface resembling a canal should be differentiated. Vertucci (1984) classified root canals according to number of canals present and their configuration into eight types [7].

Anatomic anomaly observed at first appointment should be checked for similar anomaly of tooth on the other side [8]. In the present case, radiographic image showed bilateral Vertucci type II [2-1] configuration.

Çalişkan et al. [9] studied root canal number, configuration, and ramifications of permanent teeth in Turkish population. They reported percentage of Vertucci type III [1-2-1] and type V [1-2] as 4.35 and 2.17, respectively. Alapati et al. [10] and Onay and Ungor [11] reported a maxillary right canine with type II canal configuration and Weisman [12] also reported a birooted maxillary left canine.

In the present case, the maxillary canines had an unusual root length of 31 mm which necessitated use of lengthier k flex files of 31 mm removing silicone directional stopper before use. They showed canal configuration of type II similar to that reported by Alapati et al. and Onay and Ungor. Two distinct canal orifices were located in labial/palatal direction which joined in apical third, forming a type II configuration.

Teeth with type II configuration during treatment may pose problems. The canal that is in line with the main passage is usually amenable to adequate enlarging and obturation procedures; the preparation and filling of the other canal are often extremely difficult [13].

A thorough knowledge of root canal anatomy and operator skill are essential for endodontic success. Careful clinical examination with radiographs from several different angles may lead to suspicion or identification of additional canals and leads to higher possible success [3].

## 4. Conclusion

Several variations exist in the root canal system and clinicians should be aware of the variations for complete infection removal and prevention of reinfection. Special care with careful endodontic exploration, different angle radiographs, and magnification with surgical microscope aids in detection and treatment of extra canals.
